# Protease Inhibitor Resistance Is Uncommon in HIV-1 Subtype C Infected Patients on Failing Second-Line Lopinavir/r-Containing Antiretroviral Therapy in South Africa

**DOI:** 10.1155/2011/769627

**Published:** 2010-12-02

**Authors:** Carole L. Wallis, John W. Mellors, Willem D. F. Venter, Ian Sanne, Wendy Stevens

**Affiliations:** ^1^Department of Molecular Medicine & Hematology, University of the Witwatersrand, Wits Medical School, 3B22, 3rd Floor, 7 York Road, Parktown 2193, South Africa; ^2^Division of Infectious Diseases, University of Pittsburgh, Pittsburgh, USA; ^3^Reproductive Health and HIV Research Unit, University of the Witwatersrand, Johannesburg, South Africa; ^4^Clinical HIV Research Unit, University of the Witwatersrand, Johannesburg, South Africa; ^5^National Health Laboratory Services (NHLS), Johannesburg, South Africa

## Abstract

Limited data exist on HIV-1 drug resistance patterns in South Africa following second-line protease-inhibitor containing regimen failure. This study examined drug resistance patterns emerging in 75 HIV-1 infected adults experiencing virologic failure on a second-line regimen containing 2 NRTI and lopinavir/ritonavir. Ninety six percent of patients (*n* = 72) were infected with HIV-1 subtype C, two patients were infected with HIV-1 subtype D and one with HIV-1 subtype A1. Thirty nine percent (*n* = 29) of patients had no resistance mutations in protease or reverse transcriptase suggesting that medication non-adherence was a major factor contributing to failure. Major lopinavir resistance mutations were infrequent (5 of 75; 7%), indicating that drug resistance is not the main barrier to future viral suppression.

## 1. Introduction

The South African antiretroviral roll out programme consists of a non-nucleoside reverse transcriptase inhibitor (NNRTI) based first-line regimen and a ritonavir-boosted protease inhibitor (PI) containing second-line regimen. Standard genotype analyses of first-line failure samples from South Africa have shown that the majority of patients remain susceptible to the second-line regimen of zidovudine (AZT), didanosine (ddI), and lopinavir/ritonavir (LPV/r) [1–3]. With over one million patients on antitretroviral therapy (ART) in the South African programme, a rise in second-line regimen failures is expected. Currently, little is known about treatment options after second-line failure or the frequency of protease inhibitor resistance in HIV-1 subtype C. 

Patients with HIV-1 subtype B who experience virologic failure on an initial regimen containing LPV/r infrequently have major protease (PR) mutations detected [4]. Resistance to LPV generally requires the accumulation of several mutations in the PR gene although rare, single mutations can reduce susceptibility [5, 6]. Several studies have identified naturally occurring polymorphisms in subtype C PR that may facilitate the development of PI resistance, but their clinical significance is uncertain [7, 8]. The current study assessed the occurrence of known HIV-1 drug-resistance mutations in PR and RT in patients with second-line ART failure and the remaining treatment options.

## 2. Methods

### 2.1. Patient Samples

Plasma samples from 75 patients on a failing second-line regimen (LPV/r and 2 NRTI) were sent for HIV-1 drug-resistance testing from clinics at two large state hospitals in Johannesburg, South Africa. Virologic failure was defined as having confirmed (two consecutive measurements) of plasma HIV-1 RNA greater than 5000 copies/mL. Because pretherapy samples were not available from these patients, PR sequences were compared with those from 226 LPV/r naïve patients on failing first-line ART from the same two clinics [1]. The work conducted on these samples was with the understanding and the consent of the human subjects.

### 2.2. Population Genotype Analysis

Population-based genotyping was performed using an in-house drug-resistance assay. Briefly, a 1.7 kb amplicon was generated by RT-initiated polymerase chain reaction encompassing the entire PR and partial RT-coding regions. The amplicon was sequenced using five primers that ensure bidirectional coverage from codons 1–99 of PR and codons 1–230 of RT. Sequencing was performed with either an ABI Prisms 3730 or an ABI Prism 3100-*Avant *Genetic Analyzer (Applied Biosystems, USA).

### 2.3. Data and Statistical Analyses

Sequences were assembled, manually edited using Sequencher v4.5 software (Gene codes, Ann Arbor, MI), and submitted to the ViroScore database, which uses the IAS-USA mutation list to identify HIV-1 drug resistance mutations (http://www.iasusa.org/). The frequency distribution of PR mutations was also compared in LPV/r-naïve and -exposed patients. The REGA HIV-1 subtyping tool was used to determine HIV-1 subtype of each patient sample (http://www.bioafrica.net/subtypetool/html). The chi-squared test was used to determine if the frequency of naturally occurring polymorphisms in LPV/r-naive and -exposed patients differed significantly. A *P*-value of <.01 was considered significant to adjust for the multiple comparisons across PR.

## 3. Results

A total of 75 plasma samples were available from patients experiencing virologic failure on a second-line LPV/r-based regimen (Table 1). The mean age was 34 years (IQR 29–40), 69% were female (*n* = 52), and the average time on second-line therapy was 16 months (range 4–54 months; Table 1). At the time of failure, median CD4+ T-cell count and mean HIV-1 RNA were 141 cells/mm^3^and 184,779 copies/mL, respectively. There was no difference in HIV-1 RNA or CD4 levels observed in patients with and without PI mutations (*P* = .36 and *P* = .57, resp.).

Ninety six percent of patients (*n* = 72) were infected with HIV-1 subtype C, two patients were infected with HIV-1 subtype D, and one with HIV-1 subtype A1.

Twenty nine of the 75 patients (39%) had no major mutations present in PR or the RT region examined that would confer resistance to PI, NRTI, or NNRTI, suggesting medication nonadherence to the second-line regimen. A further 19 (25%) had NNRTI resistance mutations without PI or NRTI mutations. Only five of 75 patients (7%) had major LPV resistance mutations (Table 1). The major LPV resistance mutations detected were M46I, L76V, and V82A, occurring alone or in combinations of up to 5 mutations.

**Figure 1:**
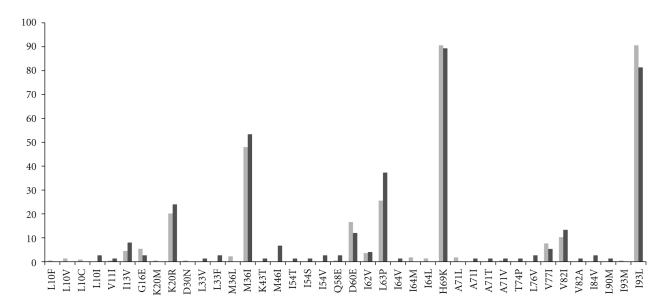
Comparison of changes from HXB2 reference between 45 lopinavir/r-exposed (light gray bars) versus 226 LPV-naïve patients (dark gray bars). Only L63P was significantly more frequent in lopinavir/r-exposed than -naïve patients (*P* = .0435).

Sixty seven (89%) patients had one or two minor lopinavir (LPV) resistance mutations (Figure 1). These minor mutations would not be expected to impact the efficacy of boosted PIs [9]. Nevertheless, we compared all 75 sequences from LPV/r-exposed patients with those from 226 LPV-naïve patients to assess if any of the minor mutations detected at failure were likely to have been selected by LPV/r. There were no statistically significant differences between LPV-naïve and -exposed patients in the frequency of changes in PR. 

**Figure 2:**
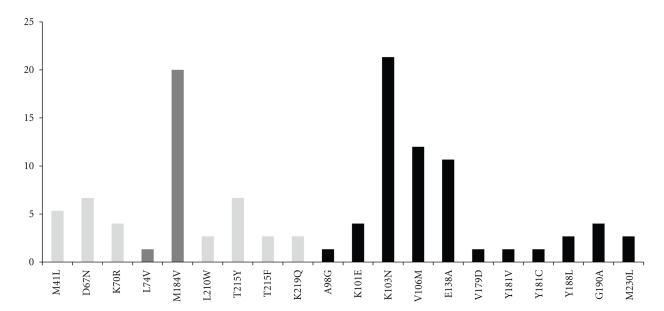
Frequency of mutations occurring in the RT region of patients failing second-line therapy. Light gray bars indicate thymidine analog mutations (TAMs), dark gray bars other NRTIs mutations, and black bars NNRTIs mutations.

Forty-one (55%) patients had mutations in the RT region only. Nineteen had mutations conferring resistance to NNRTI alone, and 22 had resistance to both NRTI and NNRTI. The most common NNRTI mutations observed were K103N (*n* = 16; 21%) and V106M (*n* = 9; 12%; Figure 2). Thirteen of the 75 patients (17%) had NNRTI resistance mutations associated with reduced susceptibility to etravirine, but only 2 of the 13 had a weighting score of greater than 2.5 predictive of a poor virologic response to etravirine. Of the 30 patients with NRTI resistance mutations, 15 had the M184V mutation, 10 had TAMs (Table 1), 1 had Q151M complex, and none had K65R. Eight percent (*n* = 6) of all patients had two or more TAMs or other resistance mutations that would cause broad NRTI cross-resistance (Q151M).

## 4. Discussion

Almost half (29 of 75 [39%]) of the patients in this study on failing second-line therapy (LPV/r and 2 NRTIs) did not have detectable resistance to NRTI, NNRTI, or PI. This suggests that medication nonadherence contributed to some of the virologic failure observed. In support of this, a recent study by Pulido et al. [10] showed that loss of viral suppression on a LPV/r regimen was linked to a low baseline CD4 count or hemoglobin levels and medication nonadherence. Nonadherence may be linked to side effects arising from the combination of ddI, AZT, or LPV/r [11], which was the most common second-line regimen prescribed (*n* = 53; 71%). Indeed, frequent toxicity was observed in a Ugandan study of second-line therapy containing LPV/r [12]. 

In patients who did have evidence of drug resistance (46 of 75 [61%]), major LPV/r mutations were infrequent (5 of 46 [11%]). Overall, only 7% (5 of 75) of the patients in this study had major LPV/r resistance mutations. The lack of accumulation of major mutations in PR is similar to that seen in other subtype C-infected patients [13]. Thus, differences in subtypes may influence the emergence of LPV resistance. In addition, our results are very similar to a recent report of 6% PI resistance in patients with viremia on second-line therapy from Soweto, South Africa [14].

Minor LPV mutations were found in 89% (67 of 75) of patients in this study, but comparison with 226 sequences from LPV/r-naïve patients revealed that all are likely to be naturally occurring subtype C polymorphisms. Several PR polymorphisms in subtype C that could affect PI susceptibility have been noted by others [8]. A recent study by Champenois and colleagues showed that there was no association between these polymorphisms and the slope of viral RNA decline or time to undetectable virus in patients receiving initial PI-based therapy, indicating that these polymorphisms probably have little impact on treatment response. Mutations outside of PR, at gag cleavage sites, can reduce susceptibility to PIs but infrequently occur without PR mutations [15]. 

Twenty seven percent of patients (20 of 75) had both NRTI and NNRTI mutations. The high percentage of K103N and V106M is likely the result of first-line failure of a NNRTI-containing regimen. M184V and TAMs were likely selected by NRTI in either the first- or second-line regimens. However, only 15% of patients had multiple TAMs or other multi-NRTI resistance mutations (Q151M) indicating that NRTI could be used again in the majority of patients. 

The mutation profiles observed indicates that several third-line options are available after second-line failure. The large majority of patients (93%) would have virus that is likely to be susceptible to other boosted PI. The 4 patients with M46I would be expected to have a decreased susceptibility to indinavir, nelfinavir, fosamprenavir, and atazanavir, and the 2 patients with L76V would likely have a reduced susceptibility to indinavir, fosamprenavir, and darunavir. The sole patient with V82A would have reduced susceptibility to indinavir. The virus with I54S would be interesting to study further as the exact effect of this mutation on PI resistance is not well understood. In addition, the second-generation NNTRI etravirine could be effective in the majority of the patients (73 of 75 [97%]), although NNRTI mutations, including etravirine resistance mutations, may have declined to undetectable levels without NNRTI exposure in the second-line regimen. Additional studies would be necessary to exclude the existence of minor populations of etravirine-resistant variants. 

## 5. Conclusion

Major LPV resistance mutations were infrequent among patients on a failing second-line regimen containing LPV/r and 2 NRTI in the South African roll-out programme. Most ritonavir-boosted PIs would be a good option for subsequent therapy, possibly in combination with etravirine and NRTIs. Alternatively, the integrase inhibitor raltegravir could be used in combination with a boosted PI although comparative data are needed. The absence of HIV-1 drug resistance in approximately half of the patients suggests that better tolerated regimens and improved adherence could achieve virus suppression without the use of new classes of antiretrovirals.

## Figures and Tables

**Table 1:** Patient characteristics and mutations at failure of second-line therapy.

Variable		Median (inter quartile range)	
	Median (inter quartile range)	No PI major mutations (*n* = 70)	PI major mutations (*n* = 5)
Age (years)	34 (29–40)		
CD4+ T-cells/mm^3^	141 (75–245)	138 (80–229)	246 (194–254)
HIV-1 RNA (copies/mL)	184,779 (8790–166,300)	61000 (15000–155000)	3260 (2200–33000)
Time on second-line (months)	16 (7–18)		

Regimens	*n*		
LPV/r, AZT, ddI	53		
LPV/r, 3TC, AZT	8		
LPV/r, 3TC, TNF	4		
LPV/r, 3TC, ABC	2		
LPV/r, AZT, d4T	2		
LPV/r, ddI, TNF	2		
LPV/r, 3TC, ddI	1		
LPV/r, 3TC, EFV	1		
LPV/r, AZT, EFV	1		
LPV/r, FTC, TNF	1		

Resistance Mutations	*n* (%)		
*NRTI mutations*	26 (35%)		
M184V	15 (20%)		
K65R	0 (0%)		
Q151M	1 (1%)		
TAMs	10 (13%)		
*NNRTI mutations*	39 (52%)		
K103N	16 (21%)		
V106M	9 (12%)		
*Any PR mutations (major and minor)*	67 (89%)		
*MajorLPV mutations*	5 (7%)		
M46I, L76V	1		
M46I	1		
L33F, I54S, V82A, I84V	1		
L33F, M46I, I54V, I84V, L90M	1		
M46I, I54V, L76V	1		
